# The Dynamic Responses of Oil Palm Leaf and Root Metabolome to Phosphorus Deficiency

**DOI:** 10.3390/metabo11040217

**Published:** 2021-04-02

**Authors:** Isiaka Ibrahim Muhammad, Siti Nor Akmar Abdullah, Halimi Mohd Saud, Noor Azmi Shaharuddin, Nurulfiza Mat Isa

**Affiliations:** 1 Laboratory of Sustainable Agronomy and Crop Protection, Institute of Plantation Studies, Universiti Putra Malaysia, Serdang, Selangor 43400, Malaysia; muhammadii108@gmail.com; 2 Department of Agriculture Technology, Faculty of Agriculture, Universiti Putra Malaysia, Serdang, Selangor 43400, Malaysia; halimi1961@gmail.com; 3 Department of Biochemistry, Faculty of Biotechnology and Biomolecular Sciences, Universiti Putra Malaysia, 43400 UPM, Serdang, Selangor 43400, Malaysia; noorazmi@upm.edu.my; 4 Department of Cell and Molecular Biology, Faculty of Biotechnology and Biomolecular Sciences, Universiti Putra Malaysia, Serdang, Selangor 43400, Malaysia; nurulfiza@upm.edu.my

**Keywords:** inorganic phosphate (Pi), P-deficiency, biochemical pathways, organic acid, lipid

## Abstract

Inorganic phosphate (Pi) starvation is an important abiotic constraint that affects plant cellular homeostasis, especially in tropical regions with high acidic soil and less solubilizable Pi. In the current work, oil palm seedlings were hydroponically maintained under optimal Pi-supply and no Pi-supply conditions for 14 days, and metabolites were measured by gas chromatography–mass spectrometry (GC–MS), from leaves and roots, after seven and 14 days of treatment, to investigate biochemical pathways in relation to P-utilizing strategy. After seven days of limited Pi, plant leaves showed increased levels of most soluble sugars, and after 14 days, the sugars’ level decrease, except for erythritol, mannose, fructose, and glucose, which showed the highest levels. Rather in root samples, there were different but overlapping alterations, mainly on sugars, amino acids, and organic acids. The leaf sample was shown to have the highest response of sugars with myo-inositol playing a vital role in the redistribution of sugars, while maltose levels increased, indicating active degradation of starch in the root. High levels of glycerol and stearate in both roots and leaves suggest the metabolism of storage lipids for cellular energy during Pi-deficient conditions.

## 1. Introduction

Orthophosphate (Pi) is an important macronutrient that plays a central role in plant metabolic processes predominantly in photosynthesis and respiration. In spite of its importance, most of the Pi in the soil is unavailable for plant uptake because of adsorption, precipitation, or conversion to organic forms [1]. Tropical and subtropical soils are the worst affected by phosphorus (P) deficiency [2] due to their acidic and low Pi characteristics [3], affecting more than 2 billion hectares [4]. Therefore, more phosphate is needed either from chemical fertilizer or through Pi fixing bacteria for healthy plant growth. Global consumption of chemical Pi for agriculture is estimated to be about 40 million metric tons per year, and this is employed to alleviate the soil inefficiency in Pi assimilation. A large amount of Pi fertilizer application and the poor assimilation by crops results in the Pi becoming immobile, or runoff into and pollute nearby surface waters [5,6]. The situation will worsen, as the global Pi reserves are projected to be exhausted by the end of this century [7,8].

Plant Pi-starvation response is a dynamic process and has a complex regulon that involves several genes responding to Pi-loss stimulus [9,10]. Pi is a vital component of nucleic acids, carbohydrates, lipids, proteins, energy transfer molecules (ATP and ADP), and Nicotinamide adenine dinucleotide phosphate (NADPH), implying that P has a central role in the molecular structures of major cellular molecules and, is indispensable for plant cell function [11,12]. When a plant is phosphate deficient, it minimizes Pi consumption [13] and utilizes Pi through the mobilization of P from different organs and cell compartments, and through the modification of metabolic processes and hydrolysis of various P-containing molecules, such as phospholipids, nucleic acids, and small phosphorylated metabolites [14,15], for the maintenance of cell metabolic homeostasis [16,17]. Transcriptomic analyses in P-deficient Arabidopsis, potato and rice have shown differential expression of several genes involved in various metabolic pathways such as carbon metabolism, photosynthesis, nitrogen assimilation, and synthesis of protein and nucleic acids, which could lead to adaptive changes in metabolite profiles [2,18,19,20].

High-throughput omics approaches, including genomics, transcriptomics, proteomics, and metabolomics have been widely used for a comprehensive characterizing of molecular mechanisms involving multiple plant physiological and biochemical processes. This provides an understanding of the various layers of the cellular responses to environmental perturbations delineating the function of biological networks [21]. Plant metabolomic studies include metabolic fingerprinting and targeted/untargeted metabolite profiling analysis. The main procedures for analysis include liquid chromatography–mass spectrometry (LC-MS), gas chromatography–mass spectrometry (GC-MS), Fourier transform-ion cyclotron resonance–mass spectrometry (FTICR-MS), capillary electrophoresis–mass spectrometry (CE-MS), and nuclear magnetic resonance (NMR) [22,23,24,25].

Recent studies on plant P-deficiency reported the abundance of di and trisaccharides in Stylosanthes roots and a significant reduction in the levels of phosphorylated metabolites like P-containing sugars, suggesting the enhancement of internal P utilization in low-Pi-stress stylo roots [26], in Lupin roots [27], and in maize [28]. Moreover, a significant increase in metabolites involved in ammonium metabolism was reported in maize leaf treated under low Pi [28,29] and also accumulation of metabolites under the influence of sucrose [11], and nitrogen-fixing bacteria in relation to P-deficiency [30].

Oil palm is a monoecious oil-producing tree belonging to the family of flowering plants, Arecaceae. It has a ubiquitous application in food ingredients, industrial produce (soap, cosmetics, and surfactant), and, recently, in biofuel prospecting. Oil palm has the highest oil output per hectare among all oil-producing plants [31]; hence, its cultivation requires a large amount of Pi supply [32], and therefore, it is significant to employ all aspects of high throughput research tools, such as genomics, transcriptomics, and metabolomics, to improve its Pi utilization efficiency.

In this study, we profiled metabolites from the leaves and roots of oil palm after two weeks of phosphate withdrawal using GC-MS analysis. To reduce and visualize the complex metabolomics datasets, multivariate statistical tools, including supervised partial least-squares-discriminate analysis (PLS-DA), and unsupervised principal component analysis (PCA), were employed. From the GC-MS profile analysis, a broad range of phosphate-deficiency responsive metabolites were quantified, and the variations in several metabolic pathways were highlighted. Moreover, to unravel the integrative biochemical networks of the oil palm in response to Pi deficiency, this work provides a better framework for understanding the mechanisms that govern cellular response to phosphate stress in oil palm leaves and roots at the molecular level.

## 2. Results

### 2.1. Plant Maintenance and Pi-Starvation Response

**Figure 1:**
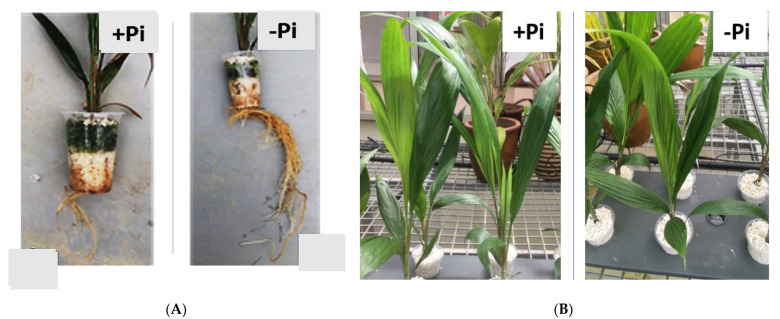
Effect of phosphate concentration on oil palm root and leaf morphology. Seedlings were maintained in hydroponic solution for 14 days. (**A**) Root morphology of oil palm supplied +Pi and no Pi developing both larger primary and secondary roots. (**B**) Leaf sample showing no difference in morphology in both +Pi and −Pi treatments.

To study P-starvation response, five-month-old oil palm seedlings were maintained in a hydroponic system either with a phosphate supply of 1.93 mM KH_2_PO_4_ (P) as control or without (NP). Leaves and roots were harvested at two different time points, after one week (seven days) and two weeks (14 days) of treatments. We observed the establishment of P-starvation in the seedlings with higher root mass in the P-deficient treatment (-Pi), as compared to the control (+Pi) (Figure 1A). Figure 1B shows leaves of oil palm in a tray treated with +Pi and with −Pi, with no significant difference in either morphology or physiology. P-deficiency symptoms after 14 days (NP2) were apparent in the root (Figure 1A). Consequently, the seedlings were extending their roots in search of more Pi in the rhizosphere. Phosphorus concentration in the root dry matter clearly showed a significant difference between treatments. Total P content in seedlings treated under NP for 14 days (NP2) reported lower P levels with 8.1 mg P/fresh weight, while roots under P supply (P2) reported higher P levels of 12.56 mg P/fresh weight. In contrast, leaf samples across all treatments did not show any significant difference in P levels. P levels in leaf after 14 days of no phosphate (NP2) and P supply (P2) conditions were reported at 8.2 mg P/fresh weight and 7.67 mg P/fresh weight respectively. (Table 1).

### 2.2. Metabolic Responses to Pi Deficiency

**Figure 2:**
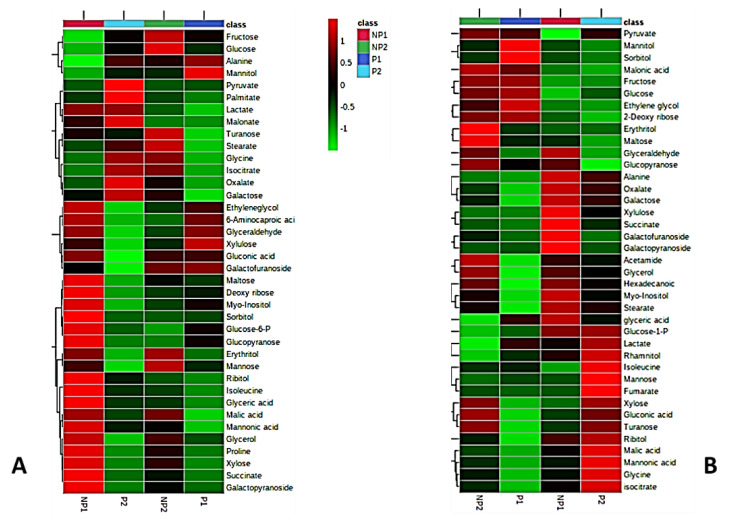
Clustered heatmaps of metabolites of leaves (**A**) and roots (**B**). Individual metabolites are displayed in rows, and nutritional status and length of treatment are represented by column. P1, seven days P-sufficient; P2, 14 days of P-sufficient; NP1, seven days without Pi-supply; NP2, 14 days without Pi-supply. Heatmap visualization is based on log10-transformed concentration of metabolites. Reddish color indicates increased levels of metabolites concentration while greenish color indicates decreased levels of metabolites concentration; *n* = 3.

A GC-MS metabolomic method was employed to compare the metabolite profile of leaves and roots of seedlings subjected to P-deficient and P-sufficient treatments at the two different time points (NP1 and NP2). Metabolites were extracted from leaf and root samples, in three replicates, for each experimental group, i.e., P-deficient and P-sufficient seedlings. A total of about 90 metabolites were detected in both leaves and roots of oil palm seedlings. After the filtering of repetitive metabolites and some noises, 38 and 39 metabolites were finally quantified from the different treatments in leaves and roots, respectively. To display a visual pattern of metabolites concentration, a heatmap was generated to show metabolite changes under P-deficiency and P-sufficient conditions at different time points. From the heatmap, leaf and root metabolic profiles were compared from seedlings subjected to either seven days (NP1) or 14 days of no P supply (NP2) to control. Increased levels of metabolite under a given treatment condition are represented by red and the decreased level is indicated by green color. Our result showed up-accumulation of 26 metabolites in the leaves of seedlings subjected to seven days (one week) of Pi-deficiency (LNP1) and 10 up-accumulation in the leaves of seedlings after 14 days (two weeks) of no P supply (LNP2). Under the Pi sufficient condition, eight metabolites showed increased levels in both the first week (LP1) and second week (LP2). Out of the 26 metabolites that showed increased levels in LNP1, 18 (turanose, maltose, ribose, myo-inositol, sorbitol, glucose-6-phosphate (Glc-6-P), glucopyranose, erythritol, mannose, isoleucine, glyceric acid, malic acid, mannonic acid, glycerol, proline, xylose, succinate, and galactopyranoside) are significantly higher than in LP1. Interestingly, only 10 metabolites from LNP2 treatment (fructose, glucose, turanose, erythritol, mannose, malic acid, glycerol, proline, xylose, and galactopyranoside) showed higher levels than in LP2. It is obvious that LNP1 seedlings resulted in more metabolites being elevated in production, compared to the controls. Some metabolites were commonly being detected in both LNP1 and LNP2 treatments but at different levels. We discovered that seven metabolites (maltose, mannonic acid, glycerol, proline, xylose, succinate, and galactopyranoside) showed higher levels in LNP1 than in LNP2. The increased levels of two metabolites (fructose and glucose) were only detected in LNP2 while five metabolites (maltose, ribose, sorbitol, isoleucine, and glyceric acid) were only detected in LNP1 (Figure 2A)

In root samples, we detected 39 metabolites from the P-deficient (RNP) and Pi-sufficient seedlings (RP). Fifteen metabolites each from seven days (RNP1) and 14 days (RNP2) days of no phosphate treatment showed up-accumulation in the root. On the other hand, six and 14 metabolites were up-accumulated in the root sample after seven days (RP1) and 14 days (RP2) of P-sufficient conditions, respectively. At the first week of no phosphate supply (RNP1), 10 metabolites’ (glyceraldehyde, glucopyranose, succinate, galactofuranoside, galactopyranoside, glycerol, hexanoic acid, myo-inositol, and stearate) levels were up-accumulated, compared to P-sufficient root (RP1) samples. In the second week of P-deficient roots (RNP2), eight metabolites (pyruvate, hexanoic acid, glycerol, erythritol, maltose, glyceraldehyde, glucopyranose, and acetamide) were significantly higher in RNP2 than in P-sufficient roots (RP2). Two of the eight metabolites (erythritol and maltose) were specifically up-accumulated in the RNP2 sample. In contrast, six metabolites (xylulose, succinate, galactofuranoside, galactopyranoside, myo-inositol, and stearate) were distinctively present only in RNP1 treatment (Figure 2B).

**Figure 3:**
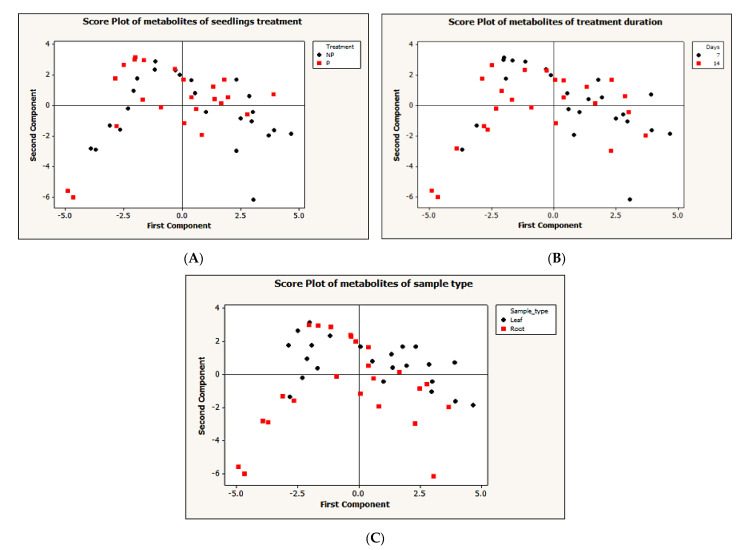
Unsupervised principal component analysis (PCA). (**A**) Two principle components (PCs) of metabolites clustering based on treatment: NP = P-deficient and N = P-sufficient seedlings (**B**) two PCs metabolites clustering based on treatment duration: seven days and 14 days (**C**) two PCs metabolites clustering based on sample type: leaf or root samples.

**Figure 4:**
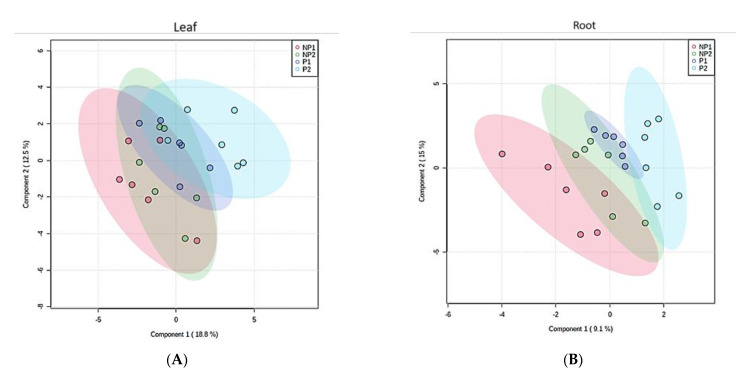
Partial-least-square discriminant analysis (PLS-DA). (**A**) Two PCs of metabolites discrimination based on treatment in the leaves. (**B**) Two PCs of metabolites discrimination based on treatment in the roots. NP1 = seven days of no Pi, NP2 = 14 days of Pi, P1 = seven days of Pi supply, and P2 = 14 days of Pi supply.

To simplify the dataset and identify a pattern, an unsupervised principal component analysis (PCA) was performed on the dataset with MINITAB 14 statistical software, using correlation (*p* < 0.05). The PCA for the first and second components generated do not show a clear pattern between different treatments (Figure 3A), treatment durations (Figure 3B) and sample types (Figure 3C). To further understand the data, we performed a supervised partial-least-squares discriminant analysis (PLS-DA). A clear discrimination between Pi-deficient and Pi-sufficient conditions was observed. Despite the two different treatment time points, both time points have clustered together according to their treatment condition (that is with or without Pi). Within the leaf sample, LNP1 and LNP2 have clustered together to discriminate from LP1 and LP2 (Figure 4A). A contrasting pattern was observed for the root sample where most RNP1 and RP2 samples grouped independently near from RNP2 and RP1, respectively (Figure 4B).

### 2.3. Metabolic Pathway Analysis in Root and Leaves during Phosphate Analysis

Pathway analyses for leaf and root samples under Pi stress were independently prepared, using the MetaboAnalyst pathway module after data normalization and scaling by log transformation and autoscaling respectively. The metabolites were mapped to the *Oryza sativa* Kyoto Encyclopedia of Genes and Genomes (KEGG) database because of the depth of rice metabolic data; it is also a monocotyledonous plant, like oil palm. The leaf (38) and root (39) metabolites were mapped to 40 (Table S1) and 36 (Table S2) pathways, respectively. After considering *p*-value (≤0.05), six metabolites (myo-inositol, D-glucose, D-galactose, D-sorbitol, D-mannose, and glycerol) from leaf samples were significantly assigned to the first 4 metabolic pathways: Inositolphosphate metabolism, Phosphatidylinositol signaling system, Ascorbate and aldarate metabolism, and Galactose metabolism (Table 2).

**Figure 5:**
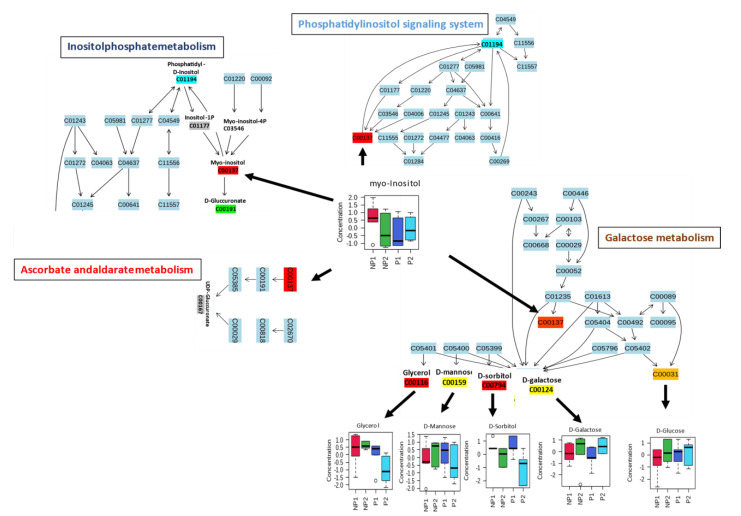
Leaf metabolic pathway responding to P-deficiency. Heavy arrows indicate multifunctionality of metabolite into other pathways.

Most of the mapped metabolites in the pathway analysis were enriched in carbohydrate metabolism. Myo-inositol metabolite has shown a ubiquitous activity in several carbohydrate metabolism pathways, with a higher increase in NP1 than NP2. It serves as a substrate in inositolphosphate metabolism, to produce D-glucuronate, which is a key precursor and interconverts uncommon less metabolizable sugars to their metabolizable forms in the pentose phosphate pathway (PPP). Meanwhile, in the ascorbate and aldarate metabolism, it is also a substrate for uridine diphosphate glucuronic acid (UDP-glucuronate) (Figure 5). D-glucuronate is a precursor of UDP-glucuronate, which can be converted to Xylulose-5-phosphate, to enter the PPP or produce vitamin C for defense [33]. Myo-inositol was found as a substrate in the production of phosphatidyl-D-inositol, which is an intermediate for phosphorylated inositol (inositol-1P, myo-inositol-4P, etc.) that has a function in the phosphatidylinositol (PIs) signaling system (Figure 5). This signaling system recruits phosphatidylinositol to control cell membrane trafficking and protein sorting [34]. Finally, myo-inositol accumulated in the galactose metabolism, which does not show any interaction with downstream metabolites in the pathway (Figure 5). The myo-inositol produced from this pathway can be used in the inositolphosphate metabolism and signaling system. Moreover, five other metabolites, namely glycerol, D-mannose, D-sorbitol, D-galactose, and D-glucose, were mapped to galactose metabolism, among which, three metabolites, i.e., D-mannose, D-sorbitol, and D-galactose, did not show any differential accumulation in the leaves of NP; meanwhile, glycerol and D-glucose showed increased accumulation in LNP1 and LNP2, respectively. Both metabolites can be utilized directly in glycolysis (D-glucose) and indirectly from the conversion of glycerol to D-glyceraldehyde [35].

Four metabolic pathways were mapped in the root samples: amino sugar and nucleotide sugar metabolism, starch and sucrose metabolism, galactose metabolism, and fructose and mannose metabolism (Table 3).

These pathways were related to eleven metabolites (myo-inositol, fructose, mannose, xylose, D-glucose-6P, D-glucose, maltose, glycerol, D-sorbitol, mannose, and α-D-galactose) from the root sample data. Like in the leaf sample, all the metabolites that were mapped in the root are carbohydrate metabolism related. In the amino sugar and nucleotide sugar metabolism, D-fructose, which was highly accumulated in NP2, serves as a precursor for the production of β-D-fructose-6P, while mannose with higher levels in RP1 was mapped as a precursor to GDP-L-galactose-1P and GDP-L-glucose through GDP-mannose intermediate.

**Figure 6:**
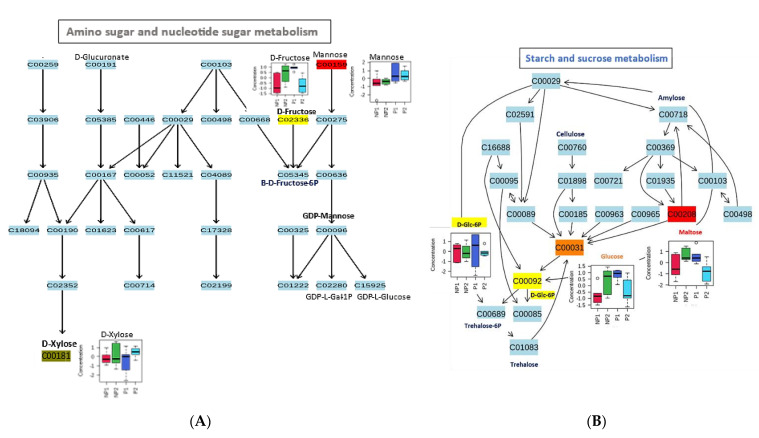

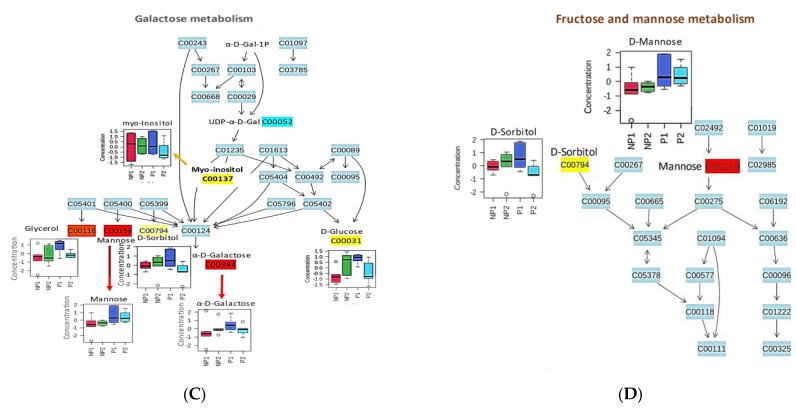
Metabolic changes in the pathway of oil palm root under phosphate stress. (**A**) Amino sugar and nucleotide metabolism. (**B**) Starch and sucrose metabolism. (**C**) Galactose metabolism. (**D**) Fructose and mannose metabolism.

Additionally, D-xylose, a pentose sugar mostly found in lignocellulose, had a high accumulation in RNP2, where it can be channeled to power the alternative pathway to glycolysis, like PPP, to keep producing energy for the plant, despite the decrease in Pi (Figure 6A).

## 3. Discussion

Plants react to Pi stress through defined molecular [36], biochemical [37,38], morphological [39], and physiological responses [27,40]. In this study, oil palm roots were found to be more sensitive to Pi-deficiency treatment than the leaves (Table 1). Withdrawal of Pi supply led to a significant decrease in P concentrations in oil palm roots, resulting in induced root morphological changes (Figure 1). The length of the lateral root was significantly extended, as is consistent with previous findings [27,38,41]. Lateral root elongation is an effort by the plant to search for more Pi in the rhizosphere [42]. However, we observed no difference in the leaf P content between P-starved and P-sufficient germinated seedlings. Earlier studies have shown sucrose (sugar) to act as a sensor in triggering the induction of P-stress transcriptional response and glucose signaling for transcription of an intricate network of genes involved in respiration and cell division [38,43]. These effects have been associated with the redistribution of sugars from leaf to root, triggering morphological changes affecting root growth due to P starvation. Metabolic responses to P status are well understood. Usually, organic acids and soluble sugars levels go down, while amino acids’ levels go up in the leaves of the Pi-deficient plants. In contrast, plant roots respond to P-deficiency by increasing the levels of organic acids, soluble sugars, and amino acids metabolic pool [28,44]. P-deficiency also leads to increased products of protein degradation and, at the same time, suppresses protein synthesis [45]. Two amino acids isoleucine and proline are involved in leaves, while alanine is involved in roots. Their levels sharply increase in their respective tissue in agreement with the previous report in oat [18], while, in contrast, glycine and isoleucine were up-accumulated in P-sufficient root sample (Figure 2).

Soluble sugars show inconsistent levels in both leaf and root samples across treatments. Eleven sugars showed an early increase at NP1 in leaves, compared to the control, and then started to diminish at NP2, while only five sugars started to respond with increased levels in leaves at NP2, compared to the control. In general, most sugars were detected in the leaves of P-deficient seedlings earlier. Furthermore, in root samples, six sugars showed an early increase in response NP1, five sugars showed increased levels at NP2, and only one sugar showed an increased level in both NP1 and NP2, compared the to control (Figure 2). Our finding is in contradiction with reports that showed decreased sugar in the leaves of P-deficient plants and lower levels of phosphorylated glycolytic intermediates (glucose-6-P) in both leaves and roots [46,47]. However, most studies frequently observed the accumulation of carbohydrates under Pi starvation [48,49] consistent with our findings in the leaf samples (Figure 2). Sucrose is synthesized in the cytosol, and glucose-1-P is a component of sucrose production that showed relatively high levels under all treatments which signifies a prevalence to channel phosphate into sucrose production which makes sucrose hardly affected by the deficiency in Pi supply. Maltose, a starch degradation product, showed high levels under Pi-deficient conditions, in roots, as a sign of photosynthetic starch reserves’ degradation even during the day [50], to make carbon supply available for the cytosol, which is in agreement with the findings in rice by Watanabe et al. (2020) [44]. Organic acids as downstream components of the glycolytic pathway [51] were found to have quite inconsistent patterns. Succinate and malate showed higher levels in leaves under NP conditions, while pyruvate and oxalate have higher levels in roots. Succinate accumulated in both leaves and roots, under insufficient P supply (Figure 2). During P-deficiency, organic acid response was tissue-specific, with increasing accumulation in the roots [47]. The levels of succinate and pyruvate increased largely under insufficient P supply [52]. In contrast, the reduced levels of some organic acids of the tricarboxylic acid cycle (TCA), including citrate, fumarate, and malate, were observed in root similar to the observation in Arabidopsis [53] and barley [29] under NP.

**Figure 7:**
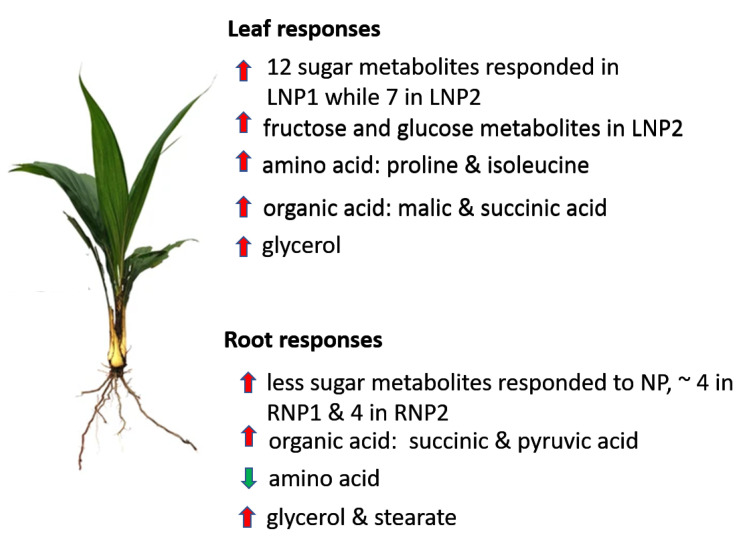
Schematic summary of metabolic response to the effect of phosphate supply in oil palm leaf and root. Red arrows represent an increase in metabolite levels, while green arrows indicate a decrease in metabolite levels.

The levels of glycerol and stearate increased under P-deficiency in the leaves (Figure 2). This may be the result of lipid degradation to source energy for the stressed plant [54]. Moreover, root tissues showed increased levels in glycerol, stearate, and palmitate. The fatty acids can be converted into metabolizable sugar by the plant. Some previous studies have shown an increase in fatty acid during limited Pi supply [54,55]. Pi-deficiency in leaves results in early accumulation of myo-inositol, which serves as an intermediate in inositol phosphate metabolism and ascorbate metabolism to synthesize D-glucuronates. D-glucuronate can be channeled to the alternative sugar metabolism pathway (PPP) [56]. Ascorbate levels usually vary between plant tissues with higher levels in leaves than in non-photosynthetic tissues like root [57]. During P-stress, myo-inositol triggers ascorbate production (Figure 5), to keep photosynthesis going [33,58]. The root metabolic pathway showed a sharp increase in the production of xylose (a pentose sugar), which is also utilized in PPP (Figure 6A) [59]. Figure 7 summarizes the metabolic responses of oil palm root and leaf to phosphate deficiency.

In conclusion, metabolomic responses by GC-MS analysis for oil palm roots and leaves during P-stress showed different but overlapping alterations, mainly on sugars, amino acids, and organic acids. The leaf sample was shown to have the highest response of sugars, with myo-inositol playing a vital role in the remobilization of sugars and maltose showing high degradation of starch in the root. There was a general decrease in protein anabolism products throughout Pii withdrawal treatment. These findings have shed more light on oil palm’s response to P-deficiency.

## 4. Materials and Methods

### 4.1. Plant Sampling and Treatment

In this study, we used three-month-old GH500 genotype (DxP) oil palm seedlings, which were procured from Sime Derby Seeds & Agricultural Services Sdn Bhd (Banting, Malaysia). Seedlings were maintained at Transgenic Greenhouse (TGH) Universiti Putra Malaysia, under controlled climatic conditions of temperature 30 °C /22 °C day/night and a photoperiod of 12 h throughout the treatment period.

Twenty-four oil palm seedlings were acclimatized for two months, at TGH, in a hydroponic Cooper 1976 solution. The Cooper 1976 medium solution contains: 1.93 mM KH2PO4, 5.77 mM KNO_3_, 4.25 mM Ca(NO_3_)_2_, 2.1 mM MgSO_4_, 0.2 mM Fe-Na_2_EDTA, 36 µM MnSO_4_, 27 µM H_3_BO_3_, 1.56 µM CuSO_4_, (NH_4_)_6_Mo_7_O_24_.4H_2_O, and 1.5 µM ZnSO_4_.7H_2_O. After acclimation, the seedlings were equally shared into phosphate (+Pi) sufficient (12 seedlings) and Pi-limited (−Pi) treatment solution (12 seedlings) for two weeks that is when the seedlings were five months old. In the Pi withdrawn treatment solution, the Pi was replaced with 0.97 mM K_2_SO_4_. Both leaves and roots were harvested weekly from four seedlings for phosphate analysis, while only three seedlings from the four were used for GC-MS analysis.

### 4.2. Total Phosphate Analysis

To estimate phosphate from oil palm leave and root, samples were prepared according to Murphy and Riley’s (1962) [60] protocol: Approximately 0.2 g of fresh plant tissues was dried at 70 °C, for two days, and placed in a porcelain crucible and baked to ash in a muffle furnace for > 3 h. The crucibles were left to cool down overnight and proceeded for P analysis. Ashes were moistened with a few drops of deionized water; then 5 mL of 100 µL of 10% HCl (*v*/*v*) and 10 mL of 20% HNO_3_ (*v*/*v*) were added to digest the sample, and the sample solution was heated on a hotplate for 1 h. After cooling, the solution was transferred into a 100 mL flask via a filter funnel, and the volume was topped up to 100 mL with deionized water. Finally, the solution was filtered for the second time, using filter paper No. 2. The final solution was sent for atomic absorption spectroscopic (AAS) for Pi measurement.

### 4.3. Metabolite Extraction

Fresh samples of leaves and roots were immediately flashed-frozen in liquid nitrogen, for storage, before analysis. Metabolite extraction was carried out according to a method by Lisec et al. (2006) [61], with minor modifications. Approximately 30 mg of frozen sample was homogenized to powder, using a chilled mortar and pestle, with liquid nitrogen. The powdered samples were transferred into a 2 mL Eppendorf tube, and 1 mL of pre-chilled 80% methanol with 10 mM of Ribitol (at −20 °C) was added to each tube. The mixture was mixed well, and further mixing at 70 °C, for 30 min, followed, using an ultrasonicator. Afterward, the tubes were spinned on a tabletop centrifugation machine, at 15,000× *g*, at RT, for 20 min. About 400 µL of the supernatant from each tube was transferred into a 1.5 mL Eppendorf tube and evaporated, using a vacuum, for more than 5 h, until the tubes were completely dry. To derivatize the samples, a two-step procedure was followed; 100 µL of methoxylamine hydrochloride in pyridine (20 mg/mL) was added to each tube, incubated for 90 min, at 37 °C, for oximation; and, subsequently, 100 µL of MSTFA was added to each tube incubated at 37 °C, for 30 min, for silylation. GC-MS analysis was carried out in three biological replicates.

### 4.4. Gas Chromatography-Mass Spectroscopy (GC-MS)

After metabolite derivatization, 0.4 µL of derivatized sample was injected in splitless mode in a Shimadzu-QP2010 Plus GC-MS system (Shimadzu, Kyoto, Japan) containing ZB-5MS 30 m × 0.25 mm ID × 0.25 µm film thickness (Madrid Avenue, Torrance, CA, USA), at a constant helium flow rate of 1 mL/min. The sample was evaporated at 250 °C, while interphase and ion source temperatures were set at 250 and 220 °C, respectively. Oven temperature was kept constant at 80 °C, for 3 min, and gradually raised to 325 °C, at a flow rate of 6 mL/min. Mass spectra of 1 scan/0.5 s with ion scanning range of 50 to 750 *m*/*z* were recorded. Metabolites were detected by blasting of each metabolite’s mass-charge ration against the mass chromatogram database of the National Institute of Standards and Technology (NIST). Each spectral area was normalized sample fresh weight and internal standard concentration; the data were filtered and sorted in an Excel sheet, before statistical analyses.

### 4.5. Statistical Analyses

Before GC-MS statistical analysis, missing values were assumed to be below the limits of detection, and these values were assigned with a minimum compound value. The relative abundance of each metabolite was log-transformed before analysis, to meet normality.

The phosphate levels’ data were analyzed in SAS software (Cary, NC, USA), for mean comparison, using least significant difference (LSD), with *p*-value (*p* ≤ 0.05). Multivariate analysis (MVA) for principal component analysis (PCA) was carried out by using MINITAB 14 (Sydney, NSW, Australia) and partial-least-squares-discriminant analysis PLS-DA by using online software Metaboanalyst (www.metaboanalyst.ca/, accessed on 18 November 2020) [62]. Heatmaps were also generated by using MetaboAnalyst online software using Pearson’s correlation and auto-scale features.

## Figures and Tables

**Table 1:** Multiple mean analysis of phosphorus levels in leaves and roots samples of oil palm maintained in hydroponics for two weeks, with and without Pi. Note: Means with the same letters are not significantly different at alpha = 0.05, using Tukey, *n* = 4.

Plant Tissue	Full Pi (mg/P/Fresh Weight)	Deprive Pi (mg/P/Fresh Weight)
Leaf	7.67 ^b^ ± 0.66	8.2 ^b^ ± 0.47
Root	12.56 ^a^ ± 1.22	8.1 ^b^ ± 0.28

**Table 2:** Pathway analysis showing phosphate activity in oil palm leaf sample.

Pathway	Total Compound	Hit	Raw p	log10(p)	Holm Adjust	FDR	Impact
Inositolphosphate metabolism	28	1	0.00456	2.34	0.178	0.0593	0.10
Phosphatidylinositol signaling system	26	1	0.00456	2.34	0.178	0.0593	0.03
Ascorbate and aldarate metabolism	18	1	0.00456	2.34	0.178	0.0593	0.00
Galactose metabolism	27	6	0.0179	1.75	0.643	0.174	0.01

**Table 3:** Pathway analysis showing phosphate activity in oil palm root sample.

Pathway	Total Compound	Hit	Raw p	log10(p)	Holm Adjust	FDR	Impact
Amino sugar and nucleotide sugar metabolism	50	3	0.0101	2.00	0.362E	0.275	0.00
Starch and sucrose metabolism	22	3	0.0224	1.650	0.784	0.275	0.54
Galactose metabolism	27	6	0.023	1.64	0.784	0.275	0.01
Fructose and mannose metabolism	20	2	0.0405	1.39	1.00	0.365	0.04

## Data Availability

The data are available on the request from corresponding author. The data are not publicly available due to their usage in the ongoing study.

## Supplementary Materials

The following are available online at https://www.mdpi.com/article/10.3390/metabo11040217/s1, Table S1: Result from pathway analysis of leaf sample, Table S2: Result from pathway analysis of root sample.
