# Supplementary material for: The Dynamic Responses of Oil Palm Leaf and Root Metabolome to Phosphorus Deficiency
Source: Metabolites. 2021 Apr 2;11(4):217. doi: 10.3390/metabo11040217 (PMC8066361; doi:10.3390/metabo11040217)
Supplement: Supplementary file 1 [file metabolites-11-00217-s001.pdf]

Table S1: Result from pathway analysis of leaf sample.

Pathway	Total Compound	Hits	Raw p	-log10(p)	Holm adjust	FDR	Impact
Inositol phosphate metabolism	28	1	4.56E-03	2.34E+00	1.78E-01	5.93E-02	0.10
Phosphatidylinositol signaling system	26	1	4.56E-03	2.34E+00	1.78E-01	5.93E-02	0.03
Ascorbate and aldarate metabolism	18	1	4.56E-03	2.34E+00	1.78E-01	5.93E-02	0.00
Galactose metabolism	27	6	1.79E-02	1.75E+00	6.43E-01	1.74E-01	0.01
Glycerolipid metabolism	21	2	6.74E-02	1.17E+00	1.00E+00	3.12E-01	0.16
Thiamine metabolism	22	2	7.78E-02	1.11E+00	1.00E+00	3.12E-01	0.00
Glycolysis / Gluconeogenesis	26	1	1.04E-01	9.83E-01	1.00E+00	3.12E-01	0.12
Cysteine and methionine metabolism	46	1	1.04E-01	9.83E-01	1.00E+00	3.12E-01	0.01
Monobactam biosynthesis	8	1	1.04E-01	9.83E-01	1.00E+00	3.12E-01	0.00
Tyrosine metabolism	18	1	1.04E-01	9.83E-01	1.00E+00	3.12E-01	0.00
C5-Branched dibasic acid metabolism	6	1	1.04E-01	9.83E-01	1.00E+00	3.12E-01	0.00
Pantothenate and CoA biosynthesis	23	1	1.04E-01	9.83E-01	1.00E+00	3.12E-01	0.00
Terpenoid backbone biosynthesis	29	1	1.04E-01	9.83E-01	1.00E+00	3.12E-01	0.00
Amino sugar and nucleotide sugar metabolism	50	3	1.13E-01	9.47E-01	1.00E+00	3.15E-01	0.00
Glycine, serine, and threonine metabolism	33	3	1.30E-01	8.85E-01	1.00E+00	3.19E-01	0.23
Glutathione metabolism	27	1	1.39E-01	8.57E-01	1.00E+00	3.19E-01	0.07
Cyanoamino acid metabolism	26	1	1.39E-01	8.57E-01	1.00E+00	3.19E-01	0.00
Carbon fixation in photosynthetic organisms	21	2	1.83E-01	7.37E-01	1.00E+00	3.97E-01	0.04
Pentose and glucuronate interconversions	17	2	2.60E-01	5.85E-01	1.00E+00	4.85E-01	0.17
Fatty acid elongation	23	1	2.73E-01	5.63E-01	1.00E+00	4.85E-01	0.00
Fatty acid degradation	37	1	2.73E-01	5.63E-01	1.00E+00	4.85E-01	0.00
Cutin, suberin, and wax biosynthesis	14	1	2.73E-01	5.63E-01	1.00E+00	4.85E-01	0.00
Valine, leucine, and isoleucine biosynthesis	22	2	3.02E-01	5.20E-01	1.00E+00	4.96E-01	0.11
Starch and sucrose metabolism	22	2	3.12E-01	5.06E-01	1.00E+00	4.96E-01	0.40
Arginine and proline metabolism	28	1	3.30E-01	4.82E-01	1.00E+00	4.96E-01	0.09
Pyruvate metabolism	22	2	3.43E-01	4.65E-01	1.00E+00	4.96E-01	0.24
Pentose phosphate pathway	19	2	3.60E-01	4.43E-01	1.00E+00	4.96E-01	0.00
Butanoate metabolism	17	2	3.62E-01	4.42E-01	1.00E+00	4.96E-01	0.00
Citrate cycle (TCA cycle)	20	3	3.77E-01	4.23E-01	1.00E+00	4.96E-01	0.09
Aminoacyl-tRNA biosynthesis	46	4	3.84E-01	4.16E-01	1.00E+00	4.96E-01	0.00
Fatty acid biosynthesis	56	3	3.94E-01	4.04E-01	1.00E+00	4.96E-01	0.01
Alanine, aspartate, and glutamate metabolism	22	3	4.14E-01	3.83E-01	1.00E+00	5.05E-01	0.00
Fructose and mannose metabolism	20	2	4.53E-01	3.44E-01	1.00E+00	5.31E-01	0.04
Selenocompound metabolism	13	1	4.63E-01	3.34E-01	1.00E+00	5.31E-01	0.00
Biosynthesis of unsaturated fatty acids	22	2	4.81E-01	3.18E-01	1.00E+00	5.36E-01	0.00
Glyoxylate and dicarboxylate metabolism	29	5	5.56E-01	2.55E-01	1.00E+00	6.03E-01	0.25
Valine, leucine, and isoleucine degradation	37	1	8.45E-01	7.33E-02	1.00E+00	8.90E-01	0.00
Sulfur metabolism	15	1	8.96E-01	4.76E-02	1.00E+00	8.96E-01	0.03
Propanoate metabolism	20	1	8.96E-01	4.76E-02	1.00E+00	8.96E-01	0.00

Table S2. Result from pathway analysis of root sample.

Pathway	Total Compound	Hits	Raw p	-log10(p)	Holm adjust	FDR	Impact
Amino sugar and nucleotide sugar metabolism	50	3	1.01E-02	2.00E+00	3.62E-01	2.75E-01	0.00
Starch and sucrose metabolism	22	3	2.24E-02	1.65E+00	7.84E-01	2.75E-01	0.54
Galactose metabolism	27	6	2.30E-02	1.64E+00	7.84E-01	2.75E-01	0.01
Fructose and mannose metabolism	20	2	4.05E-02	1.39E+00	1.00E+00	3.65E-01	0.04
Pentose phosphate pathway	19	2	7.19E-02	1.14E+00	1.00E+00	5.18E-01	0.00
Valine, leucine, and isoleucine degradation	37	1	1.38E-01	8.59E-01	1.00E+00	8.30E-01	0.00
Glycerolipid metabolism	21	2	1.67E-01	7.78E-01	1.00E+00	8.57E-01	0.16
Valine, leucine, and isoleucine biosynthesis	22	2	2.06E-01	6.86E-01	1.00E+00	9.25E-01	0.11
Glycolysis/gluconeogenesis	26	1	4.25E-01	3.71E-01	1.00E+00	9.25E-01	0.12

Cysteine and methionine metabolism	46	1	4.25E-01	3.71E-01	1.00E+00	9.25E-01	0.01
Monobactam biosynthesis	8	1	4.25E-01	3.71E-01	1.00E+00	9.25E-01	0.00
C5-Branched dibasic acid metabolism	6	1	4.25E-01	3.71E-01	1.00E+00	9.25E-01	0.00
Pantothenate and CoA biosynthesis	23	1	4.25E-01	3.71E-01	1.00E+00	9.25E-01	0.00
Terpenoid backbone biosynthesis	29	1	4.25E-01	3.71E-01	1.00E+00	9.25E-01	0.00
Aminoacyl-tRNA biosynthesis	46	3	4.36E-01	3.60E-01	1.00E+00	9.25E-01	0.00
Glutathione metabolism	27	1	4.73E-01	3.25E-01	1.00E+00	9.25E-01	0.07
Cyanoamino acid metabolism	26	1	4.73E-01	3.25E-01	1.00E+00	9.25E-01	0.00
Thiamine metabolism	22	2	4.86E-01	3.13E-01	1.00E+00	9.25E-01	0.00
Glycine, serine, and threonine metabolism	33	3	5.45E-01	2.63E-01	1.00E+00	9.25E-01	0.23
Tyrosine metabolism	18	2	6.12E-01	2.13E-01	1.00E+00	9.25E-01	0.07
Phosphatidylinositol signaling system	26	1	6.45E-01	1.90E-01	1.00E+00	9.25E-01	0.03
Ascorbate and aldarate metabolism	18	1	6.45E-01	1.90E-01	1.00E+00	9.25E-01	0.00
Arginine biosynthesis	18	1	6.49E-01	1.88E-01	1.00E+00	9.25E-01	0.00
Butanoate metabolism	17	2	6.63E-01	1.79E-01	1.00E+00	9.25E-01	0.00
Carbon fixation in photosynthetic organisms	21	2	7.30E-01	1.37E-01	1.00E+00	9.25E-01	0.04
Sulfur metabolism	15	1	7.41E-01	1.30E-01	1.00E+00	9.25E-01	0.03
Propanoate metabolism	20	1	7.41E-01	1.30E-01	1.00E+00	9.25E-01	0.00
Pyruvate metabolism	22	3	7.77E-01	1.09E-01	1.00E+00	9.25E-01	0.24
Pentose and glucuronate interconversions	17	2	7.97E-01	9.87E-02	1.00E+00	9.25E-01	0.17
Selenocompound metabolism	13	1	8.41E-01	7.54E-02	1.00E+00	9.25E-01	0.00
Alanine, aspartate, and glutamate metabolism	22	4	8.76E-01	5.73E-02	1.00E+00	9.25E-01	0.00
Glyoxylate and dicarboxylate metabolism	29	5	8.79E-01	5.61E-02	1.00E+00	9.25E-01	0.25
Inositol phosphate metabolism	28	2	9.09E-01	4.15E-02	1.00E+00	9.25E-01	0.10
Citrate cycle (TCA cycle)	20	4	9.11E-01	4.03E-02	1.00E+00	9.25E-01	0.11
Fatty acid biosynthesis	56	1	9.25E-01	3.41E-02	1.00E+00	9.25E-01	0.00
Biosynthesis of unsaturated fatty acids	22	1	9.25E-01	3.41E-02	1.00E+00	9.25E-01	0.00
